# Differential Effects of EGFR Ligands on Endocytic Sorting of the Receptor

**DOI:** 10.1111/j.1600-0854.2009.00943.x

**Published:** 2009-06-17

**Authors:** Kirstine Roepstorff, Michael Vibo Grandal, Lasse Henriksen, Stine Louise Jeppe Knudsen, Mads Lerdrup, Lene Grøvdal, Berthe Marie Willumsen, Bo van Deurs

**Affiliations:** 1 Department of Cellular and Molecular Medicine, The Panum Building, Faculty of Health Sciences, University of CopenhagenBlegdamsvej 3, DK-2200 Copenhagen, Denmark; 2 Department of Biology, University of CopenhagenOle Maaløes Vej 5, DK-2200 Copenhagen, Denmark

**Keywords:** amphiregulin, betacellulin, endocytic trafficking, epidermal growth factor, epidermal growth factor receptor, epiregulin, heparin-binding EGF-like growth factor, transforming growth factor-α

## Abstract

Endocytic downregulation is a pivotal mechanism turning off signalling from the EGF receptor (EGFR). It is well established that whereas EGF binding leads to lysosomal degradation of EGFR, transforming growth factor (TGF)-α causes receptor recycling. TGF-α therefore leads to continuous signalling and is a more potent mitogen than EGF. In addition to EGF and TGF-α, five EGFR ligands have been identified. Although many of these ligands are upregulated in cancers, very little is known about their effect on EGFR trafficking.

We have compared the effect of six different ligands on endocytic trafficking of EGFR. We find that, whereas they all stimulate receptor internalization, they have very diverse effects on endocytic sorting. Heparin-binding EGF-like growth factor and Betacellulin target all EGFRs for lysosomal degradation. In contrast, TGF-α and epiregulin lead to complete receptor recycling. EGF leads to lysosomal degradation of the majority but not all EGFRs. Amphiregulin does not target EGFR for lysosomal degradation but causes fast as well as slow EGFR recycling. The Cbl ubiquitin ligases, especially c-Cbl, are responsible for EGFR ubiquitination after stimulation with all ligands, and persistent EGFR phosphorylation and ubiquitination largely correlate with receptor degradation.

The epidermal growth factor receptor (EGFR) is a receptor tyrosine kinase involved in normal cellular growth and differentiation as well as in the pathogenesis of cancer. Seven different EGFR ligands have been identified: EGF, transforming growth factor-α (TGF-α), heparin-binding EGF-like growth factor (HB-EGF), betacellulin (BTC), amphiregulin (AR), epiregulin (EPI), and epigen (1). Several of the ligands are found in increased concentrations in human cancers, where they engage in auto- and paracrine signalling stimulating tumour progression (2–4).

Endocytic sorting is an important mechanism regulating EGFR signalling. Upon ligand-binding, EGFR is internalized and trafficked to endosomes. From endosomes, the receptor is either recycled to the cell surface, or it is transported to lysosomes for degradation (recently reviewed in (5)). The sorting to lysosomes is regulated by ubiquitination of the receptor, which in turn is dependent on the pH sensitivity of the ligand-binding. The EGF binding to EGFR is relatively stable at the pH of endosomes, so upon EGF binding EGFR is continuously ubiquitinated by the Cbl ubiquitin ligases (6) and transported to lysosomes (7,8). In contrast, TGF-α rapidly dissociates from the receptor when exposed to the low pH of endosomes (9), and the receptor becomes de-ubiquitinated and is therefore recycled back to the plasma membrane (7,10).

Whether EGFR is degraded in lysosomes or recycled to the plasma membrane is vital for the duration of the EGFR signal. Following receptor degradation, the EGFR signal will be diminished until the number of EGFRs at the plasma membrane has been reestablished by protein synthesis. In contrast, following receptor recycling, the cell is immediately able to undergo an additional round of full EGFR activation. In accordance with this, TGF-α is a more potent mitogen than EGF (11). At present, very little is known about how the remaining EGFR ligands affect endocytic sorting and degradation of EGFR, although several studies have shown that they differ substantially in their oncogenic potential (3,4,12,13).

In this study we have compared the effects of six different EGFR ligands on EGFR endocytosis, phosphorylation, ubiquitination, degradation, and recycling. All of the ligands stimulate EGFR endocytosis. However, their effects on intracellular trafficking of EGFR vary significantly, from complete recycling to complete lysosomal degradation. Whether EGFR is recycled or degraded after stimulation with the various ligands correlates well with persistent receptor phosphorylation and also with persistent Cbl ubiquitin ligase-dependent receptor ubiquitination and pH sensitivity of ligand-binding.

Based on our results and the current knowledge about the role of EGFR ligands in the pathogenesis of cancer, we propose that the oncogenic potential of the various EGFR ligands in part depends on their ability to induce receptor recycling rather than degradation.

## Results

### EGFR ligands differentially induce receptor internalization and recycling

**Figure 1:**
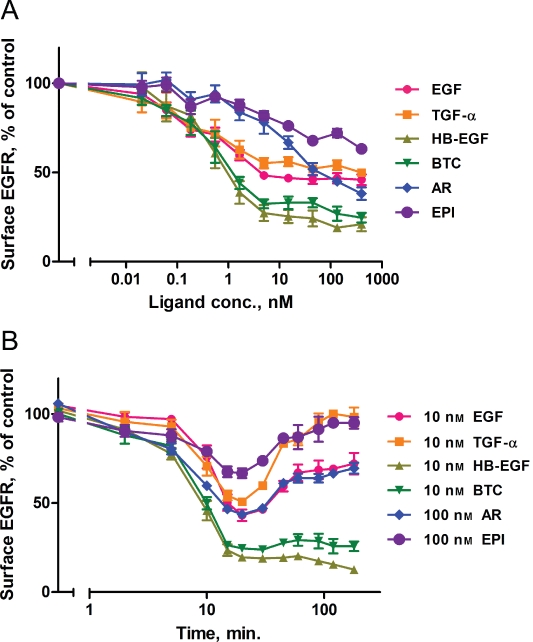
EGFR ligands differentially affect EGFR endocytosis and recycling A) EGFR internalization following stimulation with increasing concentrations of various EGFR ligands. HEp2 cells were incubated on ice with increasing concentrations of ligand for 1 h, washed, and incubated at 37°C for 15 min. Subsequently, the amount of EGFR at the cell surface was determined by FACS analysis. Data points represent mean + /− SEM for three independent experiments. B) Time–course of EGFR internalization and recycling following stimulation with different EGFR ligands. Cells were incubated on ice with 10 or 100 nm of ligands as indicated, washed, and incubated at 37°C for different time periods. The amount of EGFR present at the cell surface was determined by FACS analysis. Data points represent mean + /− SEM for four independent experiments.

To compare the ability of the different EGFR ligands to induce internalization of EGFR, we used the common approach of pre-binding ligand on ice, thereby looking at a synchronized wave of receptor internalization (7,14,15). We initially determined the incubation time necessary to obtain maximal ligand-binding. First,^125^I-EGF binding to HEp2 cells as a function of time was tested, and in Figure S1A it is seen that the equilibrium is reached after only 15 min. To investigate the binding kinetics of the other ligands to EGFR, cells were incubated with unlabelled ligand on ice for up to 2 h. Thereafter, cells were briefly washed followed by binding of ^125^I-EGF for 30 min. The level of ^125^I-EGF binding reflects unbound EGFRs. In Figure S1B it is seen that all ligands have reached maximal binding well before 1 h. Thus, to investigate EGFR internalization, HEp2 cells were incubated with increasing ligand concentrations on ice for 1 h followed by washing and 15 min chase at 37°C. Subsequently, the amount of EGFR present at the cell surface was determined by fluorescence-activated cell sorter (FACS) analysis. As seen in Figure 1A, all six ligands induced EGFR internalization. However, the degree of internalization varied between the ligands. HB-EGF and BTC were very efficient at inducing EGFR internalization. At saturating concentrations of ligand, 70–80% of cell surface EGFR was internalized upon the 15 min chase. EGF and TGF-α both induced internalization of approximately 50% of the receptors at saturating concentrations of ligand. In case of EPI and AR, the internalization did not reach saturation with the ligand concentrations used. This is in accordance with the fact that these two ligands have a lower affinity for EGFR compared with the other ligands (16,17).

The varying capability of the ligands to clear EGFR from the cell surface during a 15 min chase could be because of different trafficking properties. If a ligand induces a substantial amount of EGFR recycling to the cell surface, it will clear the cell surface for EGFR less efficiently than a ligand that does not induce receptor recycling. Such recycling can best be detected with pulse-chase experiments, because continuous incubation with ligand will cause recycled receptors to repeatedly undergo endocytosis (Figure S2). Thus, to investigate recycling, HEp2 cells were incubated with a fixed concentration of ligand on ice for 1 h, washed, and subsequently chased for the indicated period of time (Figure 1B). At the end of the chase, the amount of cell surface EGFR was quantified by FACS analysis. In case of EGF, TGF-α, HB-EGF, and BTC, 10 nm of ligand was used because this concentration induced a close to maximal EGFR internalization in the experiment shown in Figure 1A. For the lower affinity ligands EPI and AR, a concentration of 100 nm was used. Although the *in vivo* ligand concentrations have not been determined for all of the investigated ligands, it is conceivable that the concentrations used here are physiologically and pathophysiologically relevant (see discussion).

As can be seen from Figure 1B, very little EGFR is recycled to the cell surface following stimulation with HB-EGF or BTC. In contrast, close to 100% of the receptors is recycled following stimulation with either TGF-α or EPI. EGF and AR give intermediary responses, and induce recycling of approximately 50% of the internalized receptors. Thus, the six EGFR ligands have very different effects on EGFR trafficking.

### All ligands induce EGFR transport to EEA1-positive endosomes

**Figure 2:**
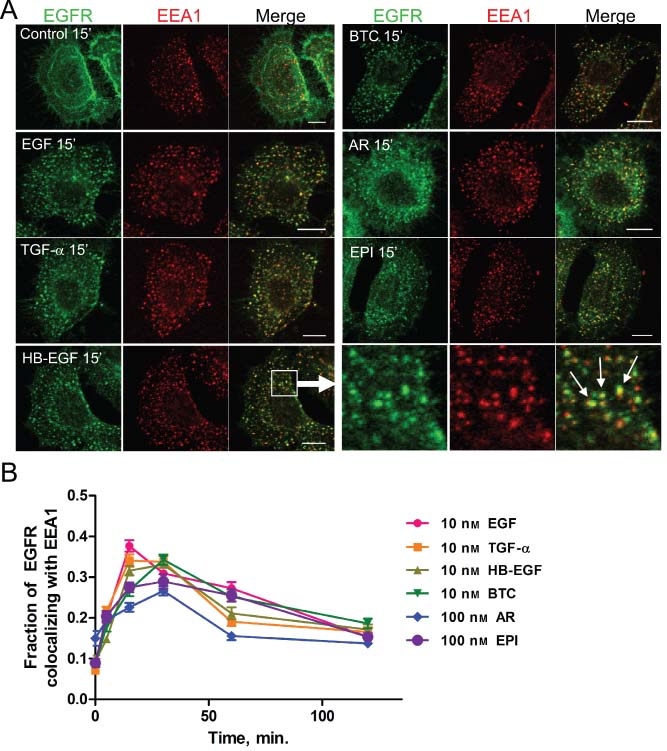
EGFR localization to early endosomes following ligand stimulation HEp2 cells were incubated on ice with 10 nm (EGF, TGF-α, HB-EGF, and BTC) or 100 nm (AR and EPI) of ligand, washed, and incubated at 37°C for different time periods. Cells were fixed and labelled for EGFR and the early endosome marker EEA1. (A) shows confocal microscopy images of representative cells after 15 min of EGFR internalization. The lower right panel shows a magnified field of the area boxed in the panel to the left. Bars, 10 μm. (B) shows a quantification of the amount of EGFR colocalizing with EEA1 in an average of 50–58 cells for each time-point + /− SEM.

It is known that EGFR is transported through EEA1 positive endosomes after stimulation with EGF (18). To further test trafficking and intracellular localization of EGFR after stimulation with the other ligands, we investigated the association of EGFR with EEA1 positive endosomes. Cells were incubated with ligand on ice for 1 h, washed, and incubated at 37°C for different time periods. They were subsequently fixed and labelled for EGFR and EEA1. Figure 2A shows images of EGFR and EEA1 following 15 min of internalization. As can be seen, all six EGFR ligands target EGFR to early EEA1-positive endosomes. Figure 2B shows image quantification of the amount of cellular EGFR associated with EEA1 positive endosomes at different time-points. All of the tested EGFR ligands induce colocalization of EGFR with EEA1, peaking after 15–30 min of internalization. However, AR is slightly less efficient at targeting EGFR to EEA1-positive endosomes than the other ligands.

### EGFR ligands vary in their potential to stimulate EGFR degradation

**Figure 3:**
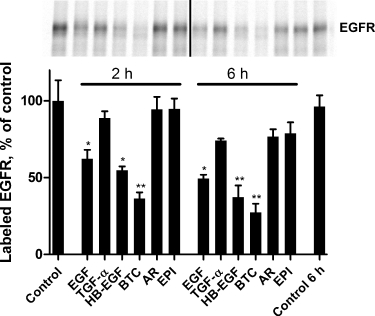
EGFR ligands differentially stimulate EGFR degradation Cells were incubated with ^35^S-methionine/cysteine for 1–2 h followed by unlabelled medium for 3 h. The cells were subsequently incubated on ice with 10 nm (EGF, TGF-α, HB-EGF, and BTC) or 100 nm (AR and EPI) of ligand, washed, and incubated at 37°C for 2 or 6 h. Cells were lysed, immunoprecipitated EGFR was separated by gel electrophoresis (upper image), and the amount of radioactive EGFR was quantified by PhosphorImager. The column bar graph shows mean + /− SEM for quantification of four independent experiments. Statistical difference from the 0 h control as determined by Student's *t*-test is indicated by stars (*: p < 0.05; ** p < 0.01).

To test how the various EGFR ligands affect receptor degradation, two different methods were applied. Cells were stimulated with ligand for 1 h on ice, washed, and chased for 0–8 h in the presence of cycloheximide to inhibit *de novo* EGFR synthesis. The cells were subsequently lysed, and the amount of EGFR determined by ELISA (Figure S3). Alternatively, to avoid the use of cycloheximide, cells were pulse-labelled with ^35^S-methionine, stimulated with ligand for 1 h on ice, washed, and incubated for 2 or 6 h at 37°C. EGFR was subsequently immunoprecipitated and the amount of ^35^S-labelled EGFR quantified by PhosphorImaging of an SDS-PAGE gel (Figure 3). As can be seen, stimulation with TGF-α, EPI, or AR does not lead to significant degradation of EGFR. Stimulation with either EGF or HB-EGF leads to degradation of 40–60% of the cellular EGFR, whereas stimulation with BTC leads to degradation of approximately 70% of the cellular EGFR.

The degradation data following stimulation with EPI, TGF- α, EGF, and BTC all correlate well with the degree of EGFR recycling induced by the ligands (compare Figure 1B and Figure 3). Interestingly, stimulation with AR does not lead to significant degradation for up to 6 h of EGFR in spite of the fact that only half of the internalized receptors are recycled within the time of the recycling experiment. One possible explanation for this is that EGFR is recycled more slowly after stimulation with AR compared with other ligands. To investigate this, we incubated cells with either EGF or AR for 1 h on ice, washed, and chased for 6 h at 37°C, 3 h longer than the recycling experiments shown in Figure 1B. We subsequently measured the amount of EGFR present at the cell surface by FACS analysis. Six hours after EGF stimulation, 60% of the initial amount of EGFR was present at the cell surface whereas 6 h after AR stimulation, the amount of EGFR on the cell surface was 80% of the initial level. This indicates that there may be some additional, slow recycling after stimulation with AR compared with EGF.

### EGFR ligands differentially induce transport of EGFR to Lamp1-positive endosomes

**Figure 4:**
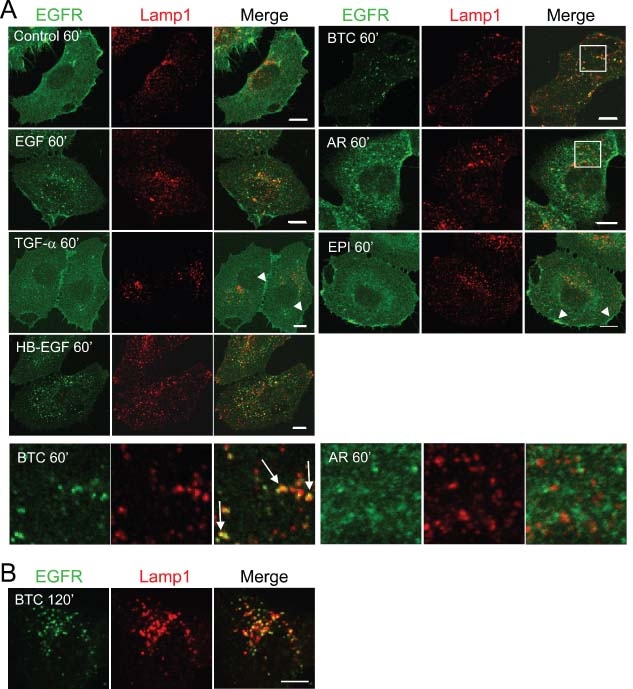
EGFR localization to lysosomes following ligand stimulation A) HEp2 cells were incubated on ice with 10 nm (EGF, TGF-α, HB-EGF, and BTC) or 100 nm (AR and EPI) of ligand, washed, and incubated at 37°C for 60 min. Cells were fixed and labelled for EGFR and the lysosomal marker Lamp1. Representative confocal microscopy images are shown. Note that in the case of TGF-α and EPI, relatively little EGFR is found inside the cell, whereas EGFR is distinct at the plasma membrane (arrow heads). The lower panel shows a magnified field of the areas boxed in the panels above. Arrows show colocalization. Bars, 10 μm. B) HEp2 cells were incubated on ice with 10 nm BTC, washed, and incubated at 37°C in the presence of 500 nm bafilomycin A1 for 120 min. Cells were fixed and labelled for EGFR and Lamp1. Note that bafilomycin A1 induces an up-concentration of EGFR in lysosomes following BTC stimulation.

We next investigated which ligands that target EGFR to lysosomes. Cells were incubated with ligand on ice for 1 h, washed, and further incubated at 37°C for different time periods. They were subsequently fixed and labelled for EGFR and Lamp1, and imaged by confocal microscopy. As can be seen from Figure 4A, after 60 min of stimulation with TGF-α or EPI a substantial amount of EGFR is present at the cell surface, which correlates well with the lack of degradation and complete recycling seen after stimulation with these ligands. Stimulation with HB-EGF, BTC, or EGF for 60 min results in some colocalization of EGFR with lysosomes. However, because the receptor is rapidly degraded once it has reached lysosomes, it is difficult to quantify the amount of EGFR trafficked to lysosomes. Indeed, when lysosomal function was inhibited by bafilomycin, most EGFR could be found in lysosomes after 120 min of stimulation with HB-EGF or BTC (not shown and Figure 4B). Following 60 min of AR stimulation, a substantial amount of EGFR appears to be located to non-lysosomal vesicles (Figure 4A). Taking into account the relatively lower colocalization with EEA1-positive compartments (Figure 2B) and the slow recycling of EGFR after AR stimulation (see above), these data indicate that AR leads to sorting of a small fraction of EGFR to an intracellular compartment which is distinct from EEA1- and Lamp-1-positive compartments and from which EGFR slowly recycles. To further characterize this compartment, we investigated colocalization of EGFR with Rab-4 and Rab-11, which are markers of recycling endosomes (19). Following 60 min of AR stimulation, EGFR partially colocalizes with both Rab-4 and Rab-11 (Figure S4). This is in agreement with a recent paper showing colocalization between EGFR and Rab-11 after AR stimulation (20).

Based on the above results, it is clear that the different EGFR ligands lead to very different trafficking and downregulation of EGFR. We therefore further investigated the molecular mechanisms underlying these differences.

### AR, HB-EGF, and BTC bind to EGFR even at very low pH

**Figure 5:**
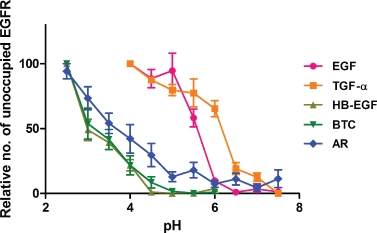
EGFR binding of HB-EGF, BTC, and AR is acid-resistant HEp2 cells were incubated with 10 nm (EGF, TGF-α, HB-EGF, and BTC) or 100 nm (AR) of unlabelled ligand on ice for 6 h followed by a brief wash with buffers of various pH. The number of ligand-free EGFRs was subsequently determined by incubation with ^125^I-EGF for 1 h on ice. The graph shows the relative increase in number of free binding sites following acid wash at different pH. Data points represent mean + /− SEM for three to six independent experiments.

It is generally accepted that TGF-α leads to EGFR recycling because the ligand dissociates from the receptor in the acidic environment in endosomes. In contrast, EGF binding to EGFR is more resistant to low pH and thus EGF leads to degradation of the receptor (9). To investigate whether there is a general correlation between receptor recycling/degradation and pH dependence of ligand-binding, cells were incubated with ligand on ice for 4 h and then briefly washed with buffers of varying pH followed by binding of ^125^I-EGF. The amount of ^125^I-EGF bound to the cells reflects the number of EGFRs from which ligand has been removed during the acid wash (Figure 5). As expected, TGF-α dissociates from the receptor at pH 6.5, whereas EGF dissociates at a slightly lower pH 5.5 (9). AR, BTC, and HB-EGF are highly acid-resistant and remain bound to the receptor at physiologically relevant pH values. It was not possible to measure the pH dependence of EPI because of the low affinity of the ligand. The data show that for EGF, TGF-α, HB-EGF, and BTC the pH sensitivity of ligand-binding correlates well with the level of receptor recycling/degradation. Surprisingly, AR binding showed high acid resistance despite the fact that this ligand induces recycling and negligible degradation of EGFR.

### EGFR ubiquitination largely correlates with receptor degradation

**Figure 6:**
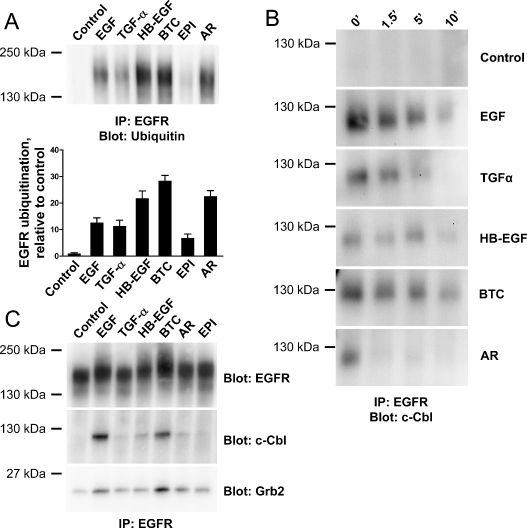
Differential effects of EGFR ligands on EGFR ubiquitination and interaction with c-Cbl A) HEp2 cells were incubated 1 h on ice with 10 nm (EGF, TGF-α, HB-EGF, and BTC) or 100 nm (AR and EPI) ligand, washed, and incubated at 37°C for 5 min. Cells were lysed in RIPA buffer, EGFR was immunoprecipitated and the amount of EGFR ubiquitination determined by western blotting for ubiquitin. The column bar graph shows the average intensity of ubiquitin signal quantified from six to seven independent experiments + /− SEM. B–C): HEp2 cells were incubated 1 h on ice with 10 nm (EGF, TGF-α, HB-EGF, and BTC) or 100 nm (AR and EPI) ligand, washed, and incubated at 37°C for 0–10 or 15 min (B and C, respectively). Cells were lysed in co-IP buffer; EGFR was immunoprecipitated; and co-precipitation of c-Cbl or Grb2 was determined by western blotting. The western blots shown are representative of three to four independent experiments.

Ubiquitination of the EGFR has been shown to target the receptor for lysosomal degradation (7). We therefore investigated to what degree the different EGFR ligands induce EGFR ubiquitination. Cells were incubated with ligand on ice for 1 h, washed, and chased for 5 min at 37°C. Subsequently, EGFR was immunoprecipitated and ubiquitination detected by western blotting. As can be seen in Figure 6A, all ligands induce ubiquitination of EGFR, albeit to a varying extent. To investigate the kinetics of EGFR ubiquitination, cells were chased for 0 to 20 min at 37°C. In Figure S5 it is seen that all ligands induce 85–100% of maximal ubiquitination after 5 min. After this time-point, ubiquitination persists to different degrees depending on the ligand. HB-EGF and in particular BTC give strong and/or persistent EGFR ubiquitination. In contrast, TGFα and EPI give low ubiquitination that is quickly lost. AR gives a strong initial EGFR ubiquitination which, however, is quickly lost. Compared with the ubiquitination seen after HB-EGF and BTC, the level of EGF-stimulated EGFR ubiquitination is low, but it is more persistent than seen after TGFα, AR and EPI (Figure 6A and Figure S5). After 60 min, the level of EGFR ubiquitination was back to background levels for all ligands (data not shown). When correlating the level and kinetics of EGFR ubiquitination (Figures 6A and S5) with that of receptor degradation, it is seen that the overall pattern of ubiquitination correlates well with the ability of the various ligands to target EGFR to lysosomal degradation.

### AR does not stimulate persistent recruitment of c-Cbl to EGFR

c-Cbl is an important ubiquitin ligase responsible for ubiquitination of EGFR (21). It can bind to EGFR either directly via phosphorylated Tyr1045 or indirectly via Grb2 (22–24). To investigate how the different EGFR ligands affect recruitment of c-Cbl to activated EGFR, cells were incubated with ligand on ice for 1 h, washed, and chased at 37°C for 0–15 min. Cells were lysed in co-immunoprecipitation (IP) buffer, EGFR immunoprecipitated, and the immunoprecipitate analysed by western blotting for EGFR, c-Cbl, and Grb2. As can be seen from Figure 6B,C, all ligands stimulate c-Cbl binding to EGFR, but the association is more prolonged after EGF and BTC stimulation than the remaining ligands. In particular, AR stimulates a transient c-Cbl binding to EGFR (Figure 6B). Based on the high ubiquitination level seen after HB-EGF, it is surprising that we found a low c-Cbl binding to EGFR after this ligand (see discussion). Like c-Cbl, Grb2 bound EGFR more efficiently following EGF or BTC stimulation for 15 min compared with the other EGFR ligands (Figure 6C). This fits well with the reports that c-Cbl primarily binds EGFR via Grb2 (25).

Recruitment of c-Cbl to the EGFR and ubiquitination of the receptor does not correlate after stimulation with AR and HB-EGF, and this could indicate that c-Cbl is not decisive for the ubiquitination of EGFR after stimulation with all ligands.

### Cbl ubiquitin ligases are important for EGFR ubiquitination after stimulation with all ligands tested

**Figure 7:**
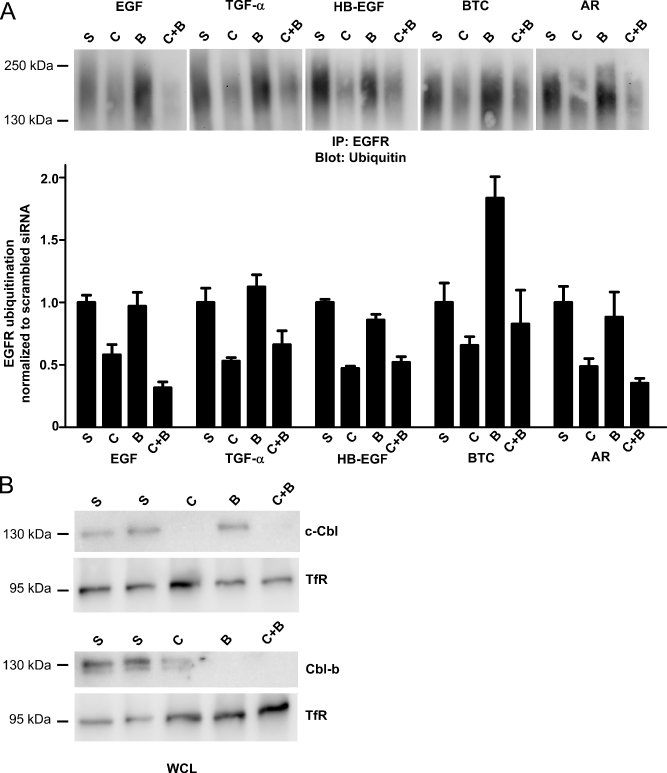
Ubiquitination of EGFR is Cbl ubiquitin ligase-dependent for all ligands HEp2 cells were transfected with scrambled siRNA or c-Cbl and/or Cbl-b siRNA twice with 48 h interval. S = scrambled siRNA, C = c-Cbl siRNA, B = Cbl-b siRNA, and C + B = c-Cbl and Cbl-b siRNA. A): 48 h after the last siRNA transfection, HEp2 cells were incubated for 1 h on ice with 10 nm (EGF, TGF-α, HB-EGF, and BTC) or 100 nm (AR) ligand, washed, and incubated at 37°C for 15 min. Cells were lysed in RIPA buffer, EGFR was immunoprecipitated, and the amount of EGFR ubiquitination determined by western blotting for ubiquitin. The column bar graph shows the average intensity of ubiquitin signal quantified from three independent experiments + /− SEM. B): 48 h after last siRNA transfection, HEp2 cells were lysed and the amounts of c-Cbl, Cbl-b, and transferrin receptor (TfR) were determined by western blotting. It is seen that c-Cbl is knocked down after treatment with c-Cbl siRNA, but c-Cbl siRNA also has an effect on Cbl-b. Cbl-b siRNA is specific for Cbl-b. TfR is used as a marker of protein levels.

To further investigate the role of Cbl ubiquitin ligases in EGFR ubiquitination, we transfected cells with small interfering RNA (siRNA) against c-Cbl and/or Cbl-b, another ubiquitin ligase reported to ubiquitinate EGFR after EGF stimulation (26), and tested how this affected ubiquitination of EGFR after stimulation with the various ligands. As seen in Figure 7, EGFR ubiquitination is partly dependent upon c-Cbl for all the investigated ligands. Knock down of Cbl-b alone does not diminish EGFR ubiquitination, but double knock down of both ubiquitin ligases does in some cases inhibit ubiquitination slightly more efficiently than c-Cbl knock down alone. This shows that Cbl ubiquitin ligases, in particular c-Cbl, are involved in EGFR ubiquitination after stimulation with all ligands.

### EGFR phosphorylation correlates with receptor degradation

**Figure 8:**
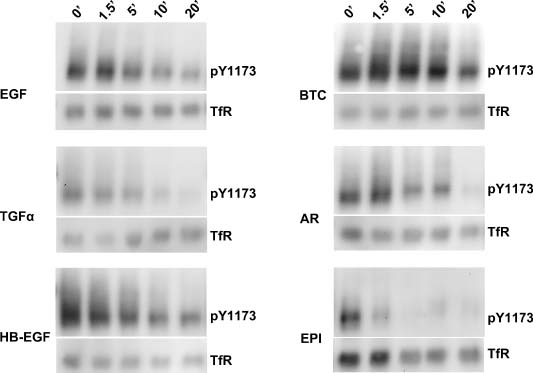
Differential effects of EGFR ligands on Tyr 1173 EGFR phosphorylation kinetics HEp2 cells were incubated 1 h on ice with 10 nm (EGF, TGF-α, HB-EGF, and BTC) or 100 nm (AR and EPI) ligand, washed, and incubated at 37°C for 0, 1.5, 5, 10 or 20 min. Cells were lysed in RIPA buffer, and the amount of EGFR phosphorylation determined by western blotting for phosphorylation on Tyr1173. TfR is used as a marker of protein levels. The western blots shown are representative of three independent experiments.

We next investigated phosphorylation of EGFR following ligand stimulation. Whole-cell lysates from cells incubated with ligand on ice for 1 h and subsequently chased at 37°C for 0–20 min were analysed by western blotting using antibodies recognizing the major phospho-tyrosine residue Tyr1173. As seen from Figure 8 all ligands stimulate EGFR phosphorylation, but EGF, BTC, and HB-EGF are more efficient at stimulating persistent EGFR phosphorylation. A similar pattern of Tyr1045, Tyr1068 and Tyr1173 phosphorylation was seen when cells were stimulated with ligand for 5 or 15 min (data not shown and Figure S6A). The low phosphorylation levels seen after TGFα, AR, and EPI stimulation are not because of limiting ligand concentration, since 10 times higher ligand concentrations do not increase the Tyr1173 phosphorylation (data not shown and Figure S6B).

To compare the site-specific phosphorylation pattern to total phosphorylation of EGFR we incubated cells with ligand on ice for 1 h followed by incubation at 37°C for 5 or 15 min (data not shown and Figure S6C), and cell lysates were subjected to IP of EGFR. The immunoprecipitates were analysed by western blotting using EGFR antibodies and antibodies recognizing general tyrosine phosphorylation. The pattern of site-specific phosphorylation on EGFR is similar to that of total phosphorylation, thus reflecting general activation.

Our data suggest that the levels of general tyrosine phosphorylation and site-specific phosphorylation of EGFR after 5–20 min correlate well with the degree of EGFR degradation following stimulation with the various ligands.

## Discussion

**Figure 9:**
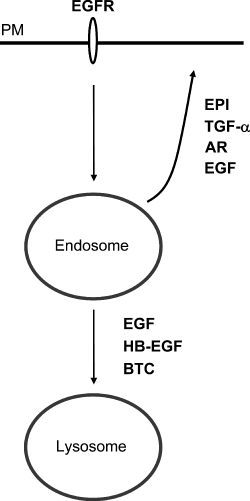
Model for EGFR trafficking after stimulation with the six ligands The model shows how EGFR is either recycled or transported to lysosomes after stimulation with the different ligands. See discussion for further details. PM, plasma membrane.

Endocytic downregulation of EGFR is an important process regulating EGFR signalling. In spite of this, very little is known about how ligands other than EGF and TGF- α affect EGFR internalization, recycling, and degradation. We show here that six different EGFR ligands differ substantially in their effects on EGFR trafficking and degradation (Figure 9).

An interesting question is whether EGFR trafficking following stimulation with the six different ligands occurs as predicted by the current model of how this is regulated, a model based solely on results of experiments with EGF and TGF-α(5). Our data show that the behaviour of EPI and BTC fits well with the model for endocytic sorting of EGFR. Accordingly, EPI stimulates endocytosis of EGFR followed by complete recycling to the cell surface, and all of the parameters measured are in accordance with this including low levels of EGFR Tyr1045 phosphorylation and low levels of EGFR ubiquitination. BTC stimulates EGFR degradation and in accordance with this, BTC leads to EGFR Tyr1045 phosphorylation, persistent c-Cbl recruitment, and EGFR ubiquitination. In contrast, the behaviour of EGFR after stimulation with HB-EGF or AR cannot be fully explained by the current model of EGFR sorting.

HB-EGF causes very efficient EGFR Tyr1045 phosphorylation, abundant EGFR ubiquitination, and EGFR degradation. However, there is only low and transitent c-Cbl recruitment to EGFR following HB-EGF stimulation in spite of the heavy Tyr1045 phosphorylation. This is surprising, since it is believed that phosphorylated Tyr1045 is an important binding site for c-Cbl (27). Interestingly, c-Cbl is recruited to EGFR in the early phases following HB-EGF stimulation, and our c-Cbl knock down experiments show that this ubiquitin ligase is necessary for full HB-EGF induced EGFR ubiquitination. This shows the necessity of testing receptor ubiquitination at a range of time-points, including very early time-points.

AR stimulation for 5 min leads to profound EGFR ubiquitination, but no receptor degradation. The lack of degradation is most likely because of the fact that the ubiquitination is rapidly lost, and therefore the receptor is not targeted to lysosomal degradation, as has previously been shown for TGF-α(7,28). In case of TGF-α, the quick loss of ubiquitination is believed to be because of the high pH sensitivity of this ligand (7,28). This is not the case for AR, which is a highly acid-resistant ligand. However, AR has a much lower affinity for EGFR than EGF, TGF-α, HB-EGF, and BTC. We therefore hypothesize that the ligand is released from EGFR in endosomes not because of pH sensitivity, but rather because of a high off-rate causing the ligand to rapidly dissociate from the receptor irrespective of the pH changes encountered during trafficking of EGFR.

Although the *in vivo* ligand concentrations have not been determined for all of the investigated ligands, it is conceivable that the concentrations used here are physiologically and pathophysiologically relevant. In case of EGF, concentrations vary strongly between different body fluids, reaching up to 8–80 nm in bile, urine, milk and prostate fluid (15,29–32). Normally, epithelia prevent these fluids from reaching EGFRs which are expressed basolaterally, but when the tight junctions of the epithelia become leaky, e.g. as a result of premalignant neoplasia, high concentrations of EGF will reach and activate EGFRs (33). Furthermore, elevated levels of EGFR ligand have been found in several cancer types (3,4,34). Although an exact ligand concentration has not been measured in tumours, due to the auto- and paracrine nature of the system, local EGFR ligand concentrations in the tumour microenvironment may reach very high levels as well.

The clinical significance of over-expression of EGFR in a multitude of cancers has been heavily investigated, and many studies have reported that high expression of EGFR in tumours is a marker of poor clinical outcome (2). Until now, the expression levels of the EGFR ligands have not been investigated to the same extent. Considering their very diverse effects on the behaviour of EGFR, it is of great importance to investigate which combinations of EGFR ligands are expressed by a tumour (3). In bladder cancer, HB-EGF, EPI, AR, and TGF-α have been associated with a lower overall survival than has BTC and EGF (4). Interestingly, apart from HB-EGF, the EGFR ligands associated with poor survival are the ligands which do not stimulate EGFR degradation and therefore allow repeated activation of EGFR.

EGFR is a well-established drug target, with several EGFR-targeting drugs on the market as well as in clinical development. However, several studies have shown that these drugs are effective on only a subset of cancer patients. One of the most important challenges is to identify the patients who will benefit from EGFR-targeting drugs prior to initiation of therapy. EGFR levels in the tumour do not appear to correlate well with clinical efficacy (35,36). In this context, it will be of great interest to investigate whether there is a correlation between EGFR ligand expression profile and sensitivity towards EGFR-targeting drugs, and whether the potential of the ligands to induce EGFR degradation correlates with clinical efficacy of EGFR-targeting drugs. In support of this hypothesis, two recent studies showed that cancer patients with tumours expressing either AR or EPI have a better response to treatment with EGFR-targeting drugs compared with patients with tumours not expressing AR or EPI (37,38). Indeed, one may envision that tumours relying heavily on EGFR signalling will tend to express ligands that do not induce EGFR degradation, and that these tumours therefore will be more sensitive to EGFR-targeted treatment. This highlights the role of ligand-induced EGFR degradation in limiting EGFR signalling, and emphasizes the importance of expanding our knowledge of how these processes are regulated.

## Materials and Methods

Unless otherwise stated, reagents were purchased from Sigma-Aldrich.

### Cell culture

HEp2 cells were maintained in DMEM supplemented with 10% FCS (Biosera), 2 mm glutamax (Invitrogen), 1 mm sodium pyruvate, 200 U/mL penicillin, and 50 ng/mL streptomycin. Cells were serum-starved in starvation medium (DMEM supplemented with 2 mm glutamax (Invitrogen), 200 U/mL penicillin, and 50 ng/mL streptomycin) 4–18 h prior to experiments.

### EGFR internalization analysis

HEp2 cells were incubated on ice 1 h with the indicated concentration of ligand (RnD Systems) in HEPES-buffered DMEM containing 0.2% BSA. Cells were washed and incubated at 37°C for the indicated period of time with or without ligand in HEPES-buffered DMEM to allow receptor internalization. Cells were subsequently placed on ice, washed 5 min with an ice-cold acidic buffer (100 mm NaCl, 50 mm glycine, pH 2.5) to remove any remaining bound ligand, neutralized with ice-cold PBS, and trypsinized on ice until detachment of cells. Trypsin was neutralized by addition of soy bean trypsin inhibitor, and the detached cells were fixed 20 min in ice-cold 2% paraformaldehyde. The amount of EGFR present at the cell surface was determined by labelling of the unpermeabilized fixed cells with an anti-EGFR antibody (clone 199.12, Thermo Fisher Scientific) directly conjugated to Alexa 488 (monoclonal antibody labelling kit, Invitrogen) followed by FACS analysis to quantify EGFR surface labelling.

### Determination of ^*125*^I-EGF binding kinetics on ice

HEp2 cells were incubated on ice with ^125^I-EGF (Perkin Elmer) for 0–60 min in HEPES-buffered DMEM containing 0.2% BSA. ^125^I-EGF was removed and the cells were washed in ice-cold medium, hydrolysed in 0.5 m KOH, and the samples counted in a gamma-counter.

### Determination of ligand-binding kinetics on ice

HEp2 cells were incubated on ice with unlabelled ligands for 0–120 min in HEPES-buffered DMEM containing 0.2% BSA. Ligand was removed and the cells were washed in ice-cold medium. To determine the number of unoccupied EGFRs following ligand-binding, cells were subsequently incubated with ^125^I-EGF for 30 min, washed, hydrolysed in 0.5 m KOH, and the samples counted in a gamma-counter. Maximal ^125^I-EGF binding was set to 0% and no ^125^I-EGF binding to 100%.

### Immunofluorescence staining and microscopy

HEp2 cells seeded in CC2-coated chamber slides were transfected with peGFP-C3-Rab4a or peGFP-C2-Rab11a using FuGene6 (Roche) or left untransfected. peGFP-C3-Rab4a and peGFP-C2-Rab11a were provided by Marino Zerial (Max Planck Institute of Molecular Cell Biology and Genetics, Dresden, Germany) and James R. Goldenring (Vanderbilt University, Nashville, Tennessee), respectively. Two to three days after seeding, cells were incubated with ligands in HEPES-buffered DMEM containing 0.2% BSA for 1 h on ice, rinsed in cold medium, and incubated for 0–120 min at 37°C in warm medium with or without 500 nm bafilomycin A1. Cells were rinsed in cold PBS and fixed in 2% paraformaldehyde. After fixation, cells were permeabilized and blocked in PBS containing 5% normal goat serum (In Vitro) and 0.2% saponin. Immunofluorescence labelling was performed using the indicated combinations of primary antibodies [mouse monoclonal anti-EGFR IgG_2A_ antibody (clone 199.12, Thermo Fisher Scientific), mouse monoclonal anti-EEA1 IgG_1_ antibody (clone 14, BD Biosciences), mouse monoclonal anti-LAMP1 IgG_1_ antibody (clone H4A3, Developmental Studies Hybridoma Bank)] and isotype specific secondary antibodies (goat anti-mouse IgG, goat anti-mouse IgG_1_, and goat anti-mouse IgG_2A_ antibodies) conjugated to Alexa 488 or Alexa 568 (Invitrogen). Microscopy was done using a Zeiss LSM 510 Meta confocal microscope, equipped with 488 and 543 nm excitation lasers, using a 63 × 1.4 NA oil immersion apochromat objective. The pinhole was set to allow acquisition of 1.0 or 1.4 μm thick confocal sections. For each condition, images of a total number of approximately 50 cells were acquired.

### Image processing and quantitative analysis

Images were sectioned into smaller images containing a single cell each using ImageJ with the MBF plug-in compilation. A binary mask defining the endosomes of the cells was made using Gaussian blur filtering (sigma = 1.0) of the EEA1 channel, and then 8-bit Otsu thresholding. The density of the EGFR signal was then integrated for the endocytic mask as well as for the entire cell. The relative amount of EGFR signal contained within the endocytic mask was calculated by dividing the signal from the mask with the total EGFR signal.

### EGFR degradation analysis using ELISA detection

HEp2 cells were incubated with ligands for 1 h on ice. After the pulse-period, cells were rinsed in cold starvation medium and then incubated in warm starvation medium containing 10 μg/mL cycloheximide at 37°C for 0–8 h. Cells were rinsed in cold PBS and scraped off in RIPA lysis buffer (1% NP40, 20 mm MOPS, 0.1% SDS, 1% Na-deoxycholate, 150 mm NaCl, and 1 mm ethylenediaminetetraacetic acid) supplemented with Protease Inhibitor Cocktail Set II and Phosphatase Inhibitor Cocktail Set III (Calbiochem). Cell debris was removed by spinning. EGFR content was then measured using an EGFR ELISA kit (RnD Systems).

### EGFR degradation analysis using ^35^S methionine/cysteine labelling

Serum-starved cells were pre-incubated in methionine- /cysteine-free DMEM supplemented with 1 mm sodium pyruvate, 2 mm glutamax, 200 U/mL penicillin, and 50 ng/mL streptomycin for 1 h. After preincubation, cells were labelled with 0.25 mCi/mL L- [^35^S]-methionine/cysteine (Mpbiomedicals) for 1–2 h. Cells were changed to starvation medium and allowed to synthesize protein for 3 h. After 3 h cells were incubated with ligands for 1 h on ice, then washed in starvation medium, and incubated in starvation medium at 37°C for 0–6 h. At the indicated times, cells were rinsed in cold PBS and then scraped off in RIPA lysis buffer supplemented with Protease Inhibitor Cocktail Set II and Phosphatase Inhibitor Cocktail Set III. Cell debris was removed by spinning at 13,000 ×***g***; the lysates were pre-cleared with protein G Dynabeads (Invitrogen), and EGFR immunoprecipitated with mouse monoclonal anti-EGFR antibody (clone 199.12) and protein G Dynabeads. The immunoprecipitates were resolved by sodium dodecyl sulfate–polyacrylamide gel electrophoresis and EGFR bands visualized by PhosphorImaging. The bands were quantified using LabVision software (Thermo Fisher Scientific), and normalized to the sum of signal detected in all EGFR-corresponding bands. The amount of EGFR present right after removal of excess ligand, the 0 h control point, was set to 100%. Whether the degradation after ligand stimulation is statistically different from the 0 h control point was tested using Student's *t*-test.

### pH sensitivity of ligand-binding

HEp2 cells were incubated on ice 4 h, with the indicated concentration of ligand in HEPES-buffered DMEM containing 0.2% BSA. Ligand was removed and the cells were washed in ice-cold medium followed by a 5-min wash with ice-cold buffers of varying pH (50 mm acetic acid, 50 mm HEPES, 50 mm NaCl, pH 2.5–7.5) followed by neutralization with HEPES-buffered DMEM. To determine the number on unoccupied EGFRs following the acid wash, cells were subsequently incubated with ^125^I-EGF on ice for 1 h, washed, hydrolysed in 0.5 m KOH, and the samples counted in a gamma-counter.

### Cbl ubiquitin ligases knock down

HEp2 cells were transfected twice with c-Cbl and/or Cbl-b siRNA (sc-29949 and sc-29950, Santa Cruz Biotechnology) or scrambled siRNA (AM4635, Ambion) using Lipofectamine2000 (Invitrogen). Transfections were done with 48 h interval and evaluation of EGFR ubiquitination and Cbl ubiquitin ligases knock down was done 48 h after the second transfection. c-Cbl and Cbl-b knock downs were evaluated with western blotting using a rabbit polyclonal anti-c-Cbl antibody (sc-170, Santa Cruz Biotechnology), a mouse monoclonal anti-Cbl-b antibody (G-1, Santa Cruz Biotechnology), or a mouse monoclonal anti-transferrin antibody (H68.4, Zymed) and horseradish peroxidase (HRP)-conjugated secondary antibodies.

### Ubiquitination and total phosphorylation of EGFR

HEp2 cells were incubated on ice 1 h with the indicated concentration of ligand in HEPES-buffered DMEM containing 0.2% BSA. Cells were washed and incubated at 37°C in HEPES-buffered DMEM for the indicated periods of time, placed on ice and washed with ice-cold PBS before lysis in RIPA buffer containing 10 nm n-ethylmaleimide, Benzonase (Novagen, Merck Chemicals Ltd.), Protease Inhibitor Cocktail Set II, and Phosphatase Inhibitor Cocktail Set III. Cell debris was removed by spinning, and EGFR was immunoprecipitated from the lysate using a mouse monoclonal anti-EGFR antibody (clone 199.12) and protein G sepharose beads or protein G Dynabeads (Invitrogen). The precipitate was subject to western blotting using a mouse monoclonal anti-ubiquitin antibody (clone P4D1, Santa Cruz Biotechnology), a mouse monoclonal anti-tyrosine phosphorylation antibody [clone 4G10, Upstate (Millipore)] or a sheep polyclonal anti-EGFR antibody (Fitzgerald Antibodies) and HRP-conjugated secondary antibodies (DAKO). Imaging and quantification of bands were done using ECL (Amersham Biosciences) and an AutoChemi system darkroom (UVP) equipped with LabVision software (Thermo Fisher Scientific).

### Co-immunopreciptation of EGFR and c-Cbl and Grb2

HEp2 cells were incubated on ice 1 h with the indicated concentration of ligand in HEPES-buffered DMEM containing 0.2% BSA. Cells were washed and incubated at 37°C in HEPES-buffered DMEM for the indicated periods of time, placed on ice and washed with ice-cold PBS before lysis in co-IP buffer (0.5% Triton X-100, 50 mm KCl, 50 mm NaCl, 50 mm Tris-HCl, pH 7.5,) containing 10 nm n-ethylmaleimide, Protease Inhibitor Cocktail Set II, and Phosphatase Inhibitor Cocktail Set III. Cell debris was removed by spinning at 13,000 ×***g***, and EGFR was immunoprecipitated from the lysate using a mouse monoclonal anti-EGFR antibody (clone 199.12, Thermo Fisher Scientific) and protein G dynabeads (Invitrogen). The precipitate was subject to western blotting using a mouse monoclonal anti-c-Cbl antibody (clone C-15, Santa Cruz Biotechnology), a rabbit anti-Grb2 antibody (Cell Signaling Technology) or a sheep polyclonal anti-EGFR antibody (Fitzgerald Antibodies) and HRP-conjugated secondary antibodies.

### Detection of Tyr1045, Tyr1068, Tyr1173 phosphorylation of EGFR

HEp2 cells were incubated on ice 1 h with the indicated concentration of ligand in HEPES-buffered DMEM containing 0.2% BSA. Cells were washed and incubated at 37°C in HEPES-buffered DMEM for the indicated periods of time, placed on ice and washed with ice-cold PBS before lysis in RIPA buffer containing Protease Inhibitor Cocktail Set II and Phosphatase Inhibitor Cocktail Set III. Cell debris was removed by spinning at 13,000 ×***g*** and lysates were subject to western blotting using mouse monoclonal anti-transferrin antibody (H68.4, Zymed), mouse monoclonal phosphospecific anti-EGFRpTyr1045 antibody (clone 11C2, NanoTools), mouse monoclonal phosphospecific anti-EGFRpTyr1068 antibody (clone 15A2, NanoTools), mouse monoclonal phosphospecific anti-EGFRpTyr1173 antibody [clone 9H2, Upstate (Millipore)] or a sheep polyclonal anti-EGFR antibody (Fitzgerald Antibodies) and HRP-conjugated secondary antibodies.

## Supporting Information

**Figure S1: Ligands have reachedmaximal binding to EGFR well before 1 h.** A): HEp2 cells were incubated on ice with 10 nM of EGF (1 nM ^125^IEGF and 9 nM unlabelled EGF) for 0–60 min and washed. Lysates were counted in a gamma-counter. The graph shows the increase in bound ^125^I-EGF. Data points represent mean +/− SEM for five independent experiments. B): HEp2 cells were incubated on ice with unlabelled ligands for 0–120 min and washed. The number of unoccupied EGFRs following the unlabelled ligand incubation was determined by incubation with 125IEGF. The graph shows the increase in unlabelled ligands bound. This indirect method of measuring ligand-binding was used because some of the EGFR ligands also bind to ErbB4, and it is therefore not possible to separately analyse binding to EGFR using directly labelled ligands.Maximal ^125^I-EGF binding was set to 0% and no ^125^I-EGF binding to 100%.

**Figure S2: EGFR trafficking after ligand stimulation is best monitored by pulse-chase experiments.** Time-course of EGFR internalization and recycling following stimulation with EGF or TGF-α either as a pulse or continuously. Cells were incubated on ice with 10 nM of ligands as indicated, washed, and incubated at 37°C for different time periods with or without ligand in the medium. The amount of EGFR present at the cell surface was determined by FACS analysis. Data points represent mean +/− SEM for three independent experiments.

**Figure S3: EGFR ligands differentially affect EGFR degradation.** HEp2 cells were incubated on ice with 10 or 100 nM of ligands as indicated, washed, and incubated at 37°C for different time periods in the presence of 10 μM cycloheximide to block protein synthesis. Cells were lysed, and the amount of EGFR was determined by ELISA. Data points represent mean +/− SEM for three to seven independent experiments.

**Figure S4: EGFR localization to Rab-4 and Rab-11 positive endosomes following AR stimulation.** HEp2 cells were transfected with GFP-Rab- 4 or GFP-Rab-11, and incubated on ice with 100 nM AR, washed, and incubated at 37°C for 60 min. Cells were fixed and labelled for EGFR. Representative confocal microscopy images are shown. Bars, 10 μm.

**Figure S5: Differential effects of EGFR ligands on EGFR ubiquitination kinetics.** HEp2 cells were incubated 1 h on ice with 10 nM (EGF, TGF- α, HB-EGF, and BTC) or 100 nM (AR and EPI) ligand, washed, and incubated at 37°C for 0–20 min. Cells were lysed in RIPA buffer, EGFR was immunoprecipitated, and the amount of EGFR ubiquitination determined by western blotting for ubiquitin. Note that the overall intensity of the different western blots should not be compared. To compare the effect on ubiquitination levels between the different ligands, see Figure 6A. The graphs show the average intensity of ubiquitin signal quantified from three independent experiments +/− SEM.

**Figure S6: Differential effects of EGFR ligands on EGFR phosphorylation.** A) HEp2 cells were incubated for 1 h on ice with 10 nM(EGF, TGF-α, HB-EGF, and BTC) or 100 nM (AR and EPI) ligand, washed, and incubated at 37°C for 15 min. Cells were lysed in RIPA buffer and the amount of EGFR phosphorylation on Tyr1045, Tyr1068, and Tyr1173 was determined by western blotting. The western blots shown are representative of four independent experiments. B) HEp2 cells were incubated for 1 h on ice with ligands in the following concentrations: 10 nM (EGF, TGF-α, HB-EGF, and BTC), 100 nM (AR and EPI), or the 10 times higher concentrations for TGFα, AR, and EPI (100 nM TGFα, 1 αM AR and 1 αM EPI), Then cells were washed, and incubated at 37°C for 15 min. Cells were lysed in RIPA buffer and the amount of EGFR phosphorylation on Tyr1173 was determined by western blotting. The western blots shown are representative of three independent experiments. The column bar graph shows the average intensity of phosphorylation signal quantified from three independent experiments +/− SEM. C): HEp2 cells were incubated for 1 h on ice with 10 nM (EGF, TGF-α, HB-EGF, and BTC) or 100 nM (AR and EPI) ligand, washed, and incubated at 37°C for 15 min. Cells were lysed in RIPA buffer, EGFR was immunoprecipitated, and the amount of EGFR ubiquitination determined by western blotting for phosphorylated tyrosine. The column bar graph shows the average intensity of phosphorylation signal quantified from four independent experiments +/− SEM.
