# Supplementary figures and images for: Differential Effects of EGFR Ligands on Endocytic Sorting of the Receptor
Source: Traffic. 2009 Jun 17;10(8):1115–27. doi: 10.1111/j.1600-0854.2009.00943.x (PMC2723868; doi:10.1111/j.1600-0854.2009.00943.x)

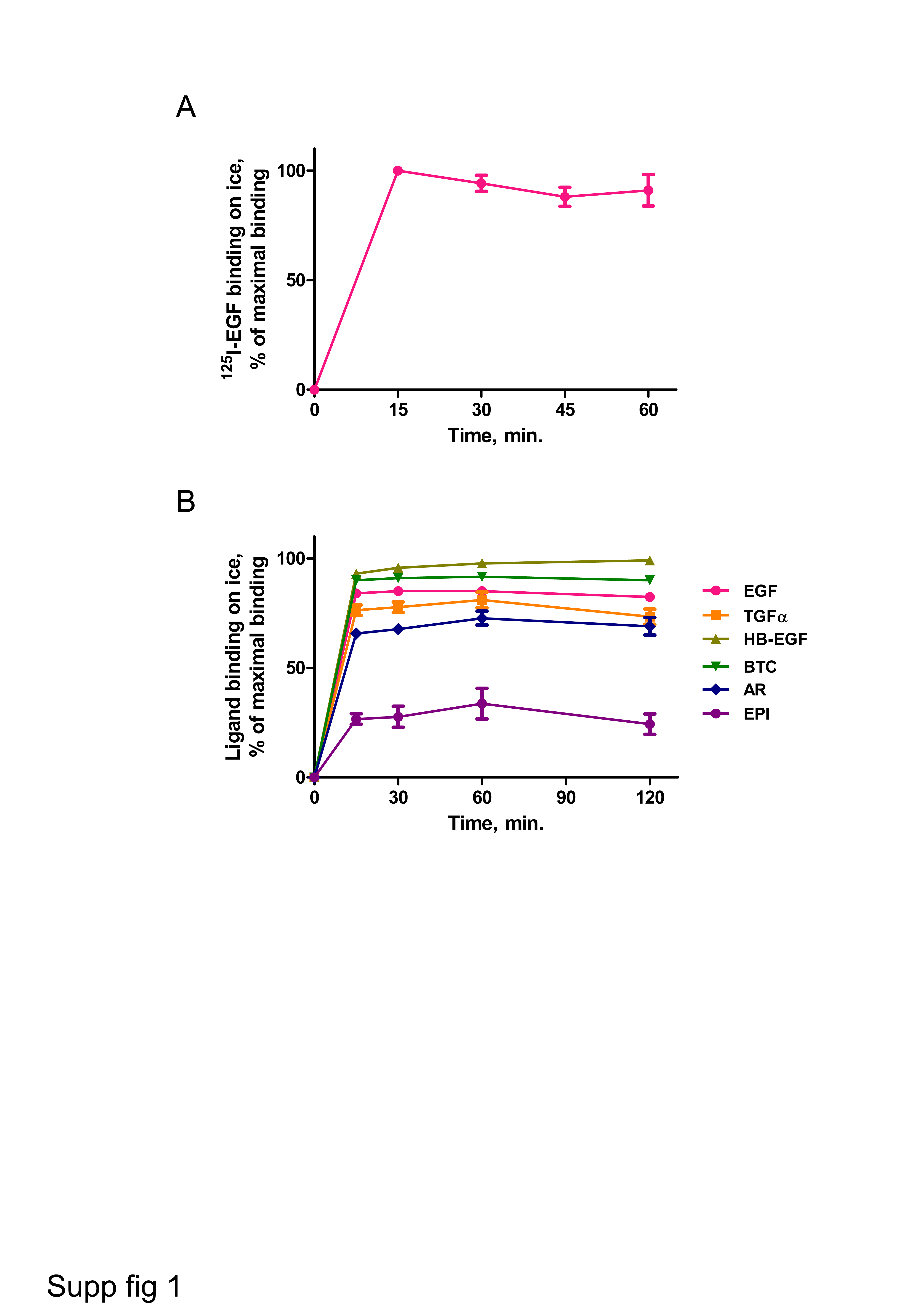

Supplement: Supplementary file 1 [file tra0010-1115-SD1.tif]

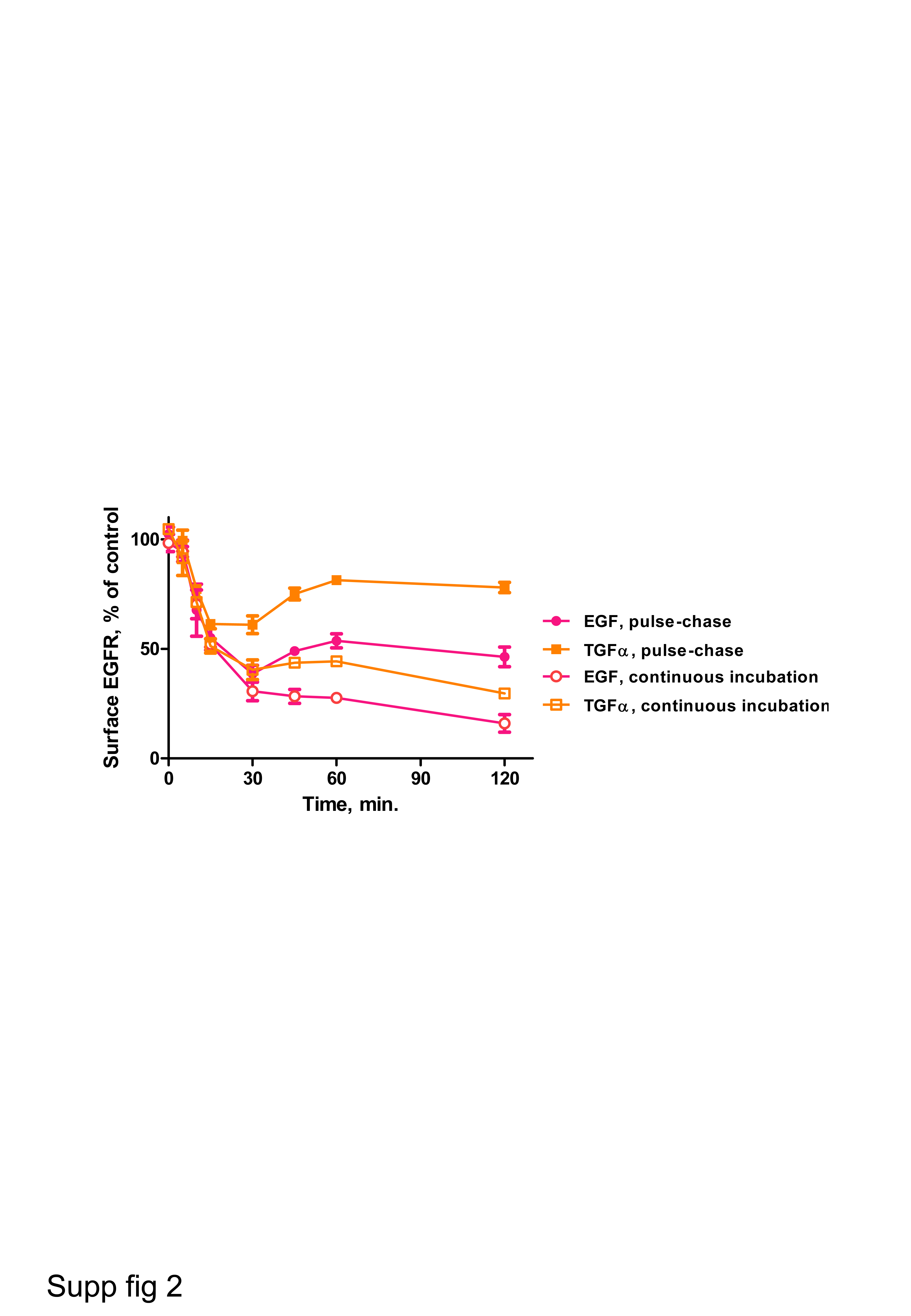

Supplement: Supplementary file 2 [file tra0010-1115-SD2.tif]

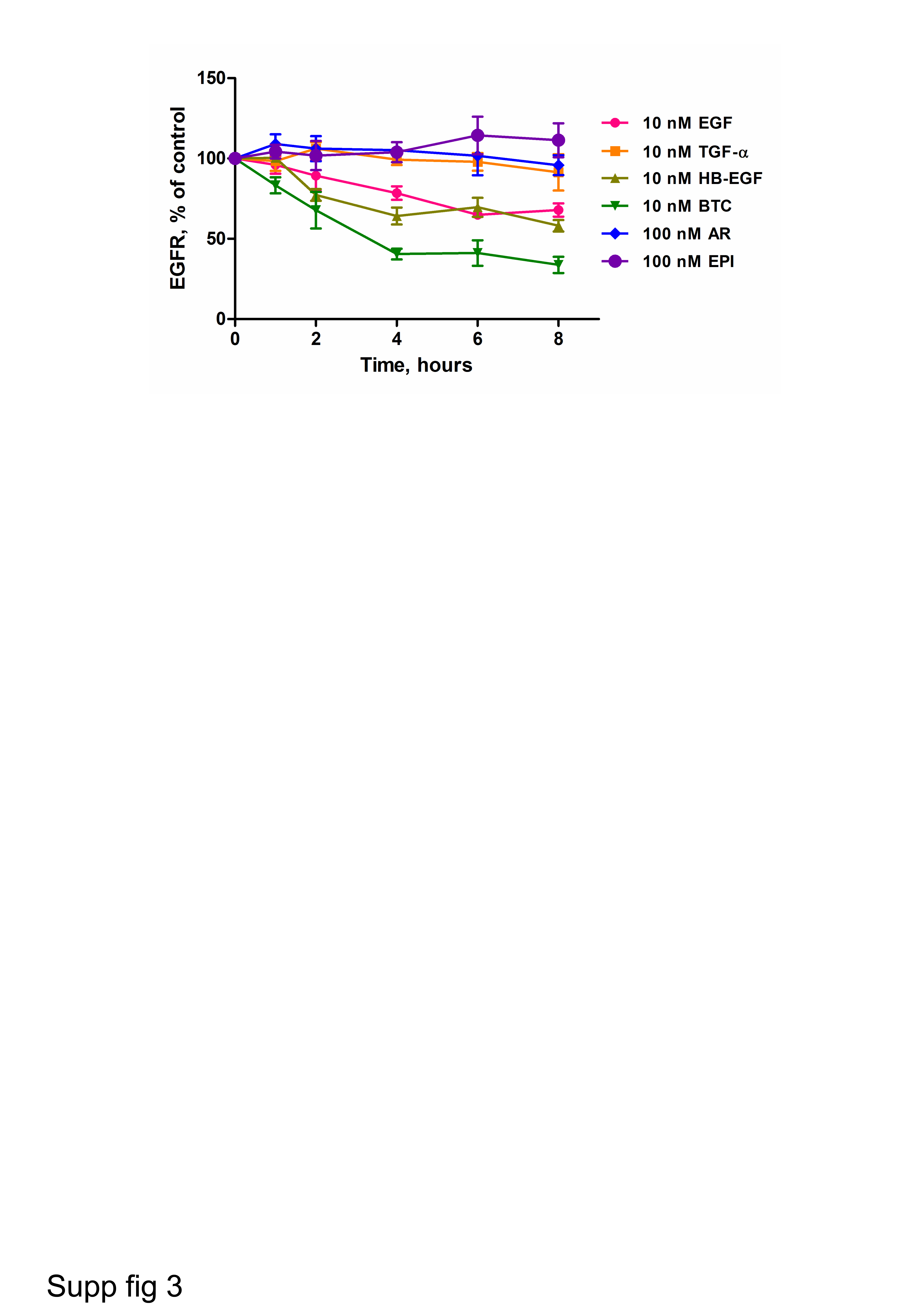

Supplement: Supplementary file 3 [file tra0010-1115-SD3.tif]

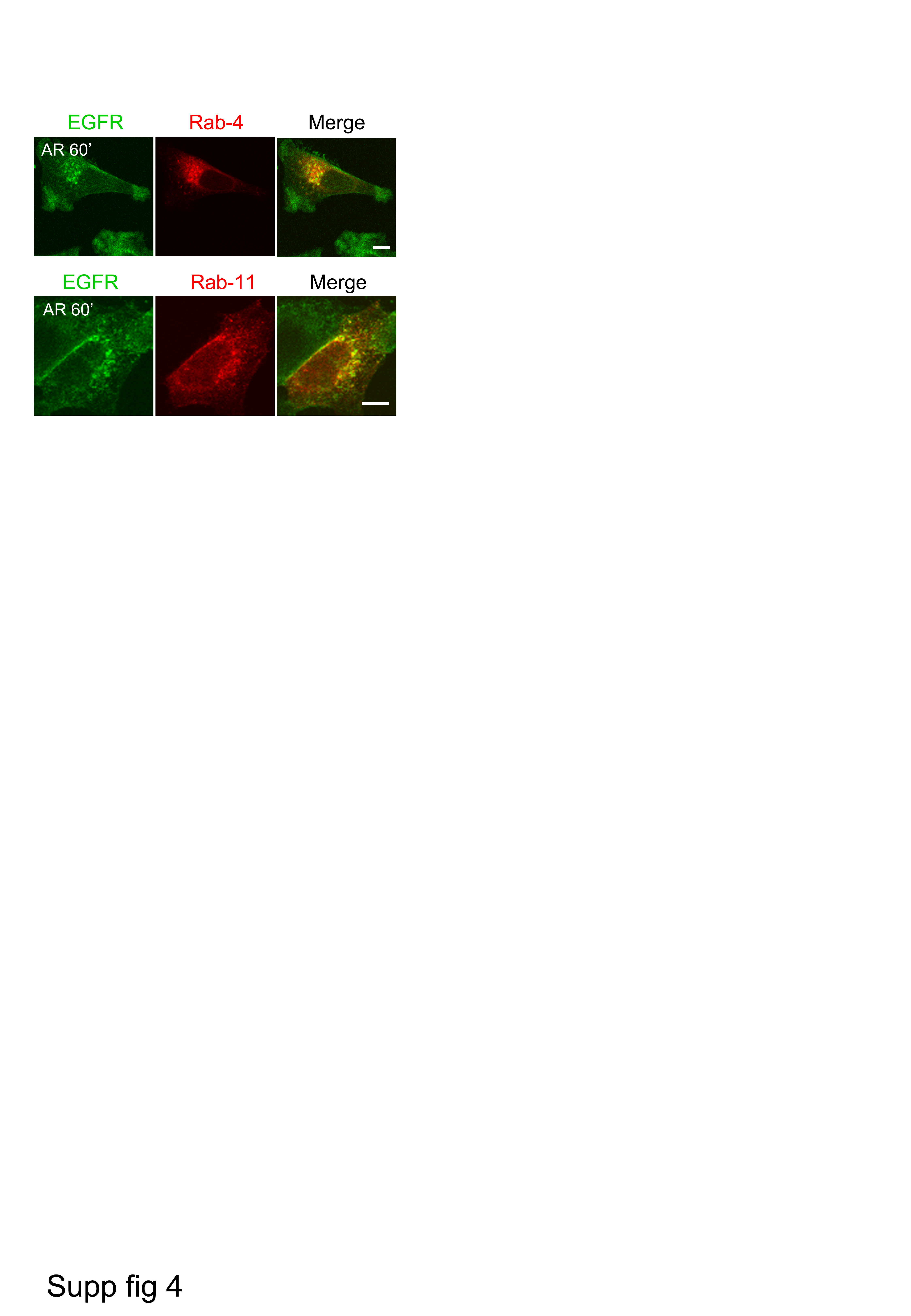

Supplement: Supplementary file 4 [file tra0010-1115-SD4.tif]

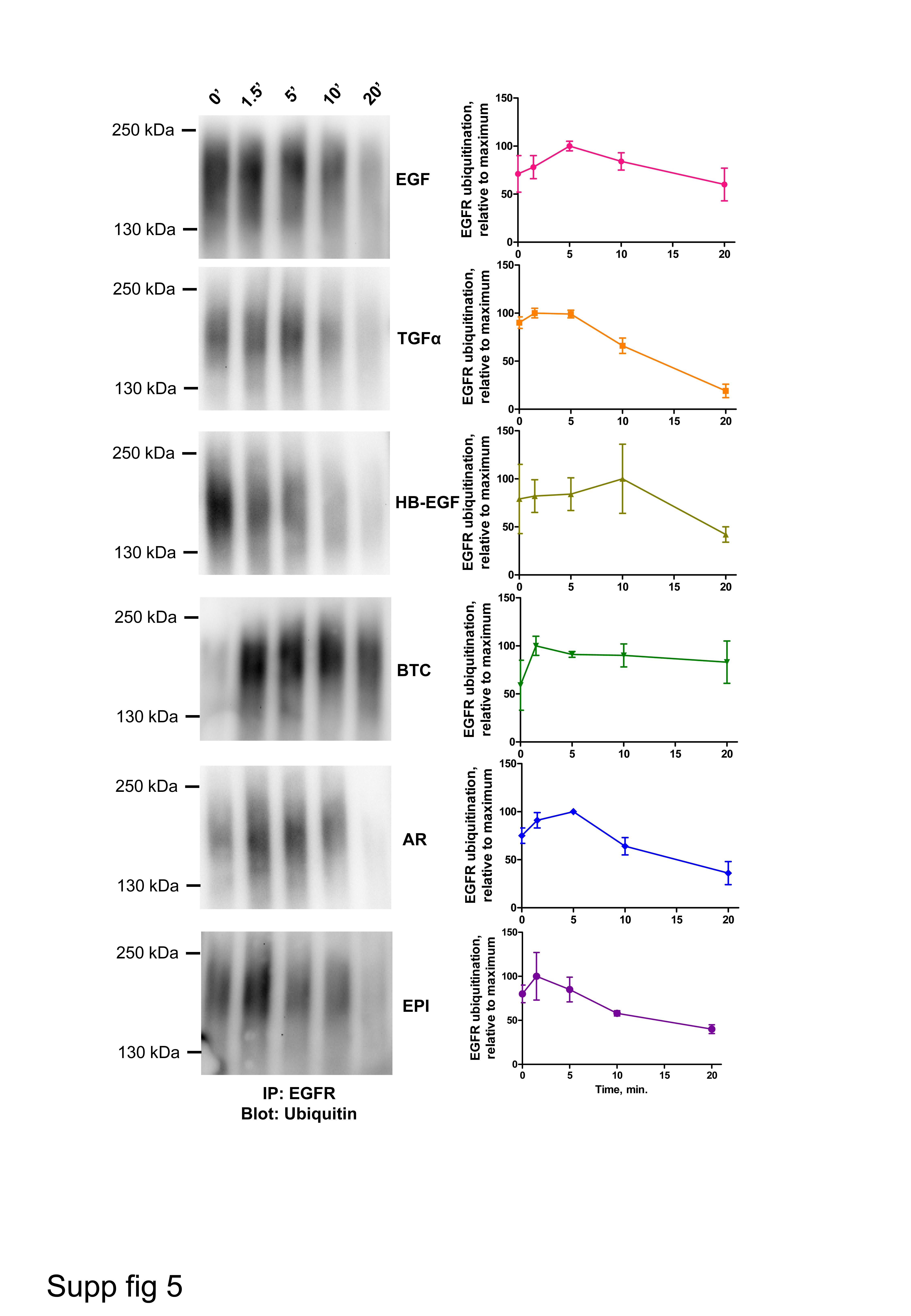

Supplement: Supplementary file 5 [file tra0010-1115-SD5.tif]

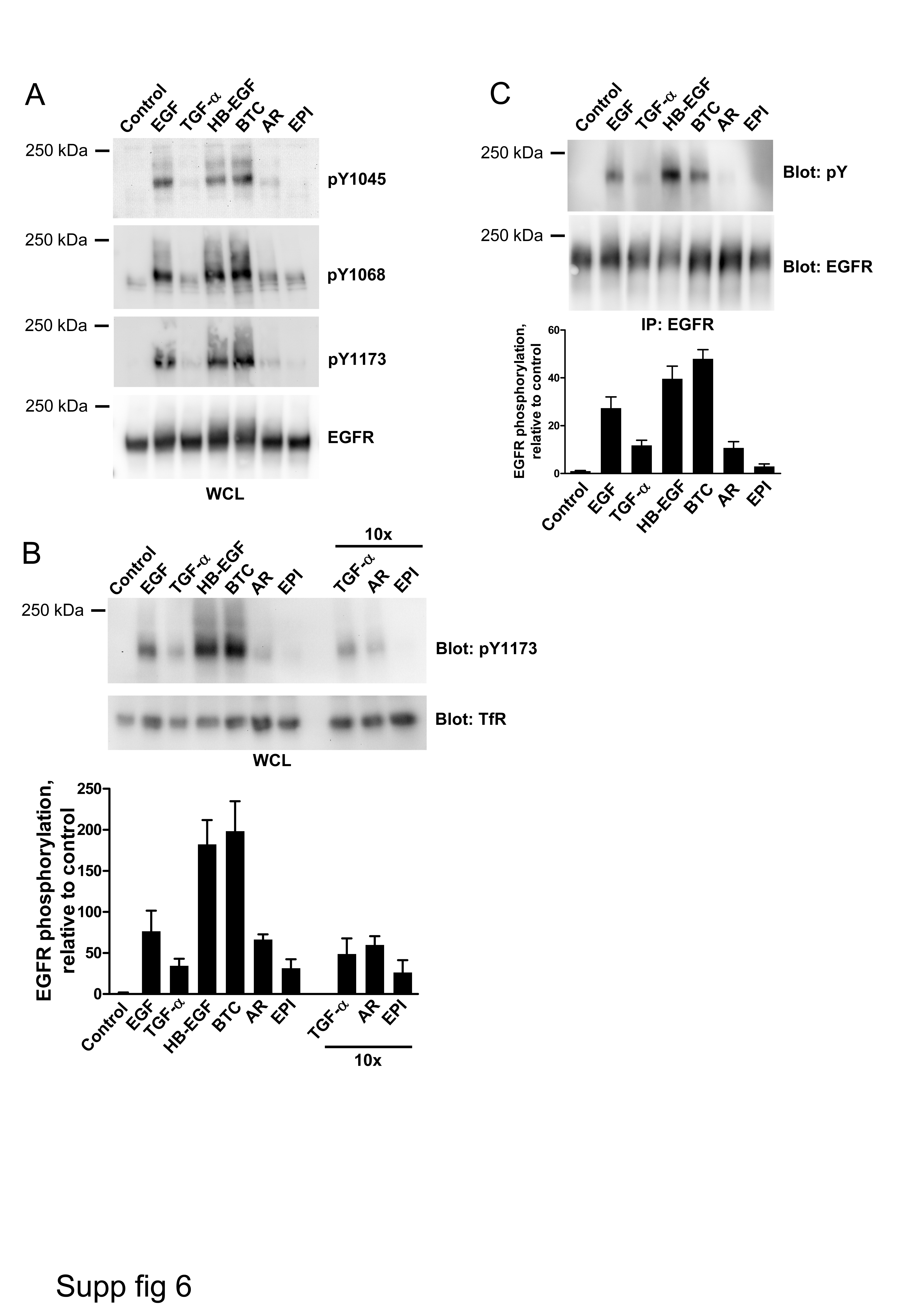

Supplement: Supplementary file 6 [file tra0010-1115-SD6.tif]
